# Protection from noise-induced cochlear synaptopathy by virally mediated overexpression of NT3

**DOI:** 10.1038/s41598-019-51724-6

**Published:** 2019-10-25

**Authors:** Ken Hashimoto, Tyler T. Hickman, Jun Suzuki, Lingchao Ji, David C. Kohrman, Gabriel Corfas, M. Charles Liberman

**Affiliations:** 1 Department of Otolaryngology–Head and Neck Surgery, Harvard Medical School, Boston, MA USA; 2 Eaton-Peabody Laboratories, Massachusetts Eye & Ear, Boston, MA USA; 3 Department of Otorhinolaryngology-Head and Neck Surgery, Tohoku University Graduate School of Medicine, Sendai, Miyagi Japan; 4 Kresge Hearing Research Institute, University of Michigan, Ann Arbor, MI USA; 5 Department of Otolaryngology-Head and Neck Surgery, University of Michigan, Ann Arbor, MI USA

**Keywords:** Cochlea, Gene therapy, Regeneration and repair in the nervous system

## Abstract

Noise exposures causing only transient threshold shifts can destroy auditory-nerve synapses without damaging hair cells. Here, we asked whether virally mediated neurotrophin3 (NT3) overexpression can repair this damage. CBA/CaJ mice at 6 wks were injected unilaterally with adeno-associated virus (AAV) containing either NT3 or GFP genes, via the posterior semicircular canal, 3 wks prior to, or 5 hrs after, noise exposure. Controls included exposed animals receiving vehicle only, and unexposed animals receiving virus. Thresholds were measured 2 wks post-exposure, just before cochleas were harvested for histological analysis. In separate virus-injected animals, unexposed cochleas were extracted for qRT-PCR. The GFP reporter showed that inner hair cells (IHCs) were transfected throughout the cochlea, and outer hair cells mainly in the apex. qRT-PCR showed 4- to 10-fold overexpression of NT3 from 1–21 days post-injection, and 1.7-fold overexpression at 40 days. AAV-NT3 delivered prior to noise exposure produced a dose-dependent reduction of synaptopathy, with nearly complete rescue at some cochlear locations. In unexposed ears, NT3 overexpression did not affect thresholds, however GFP overexpression caused IHC loss. In exposed ears, NT3 overexpression increased permanent threshold shifts. Thus, although NT3 overexpression can minimize noise-induced synaptic damage, the forced overexpression may be harmful to hair cells themselves during cochlear overstimulation.

## Introduction

In both noise-induced and age-related hearing loss, the synaptic connections between cochlear nerve fibers and their peripheral targets, the hair cells (HC), are more vulnerable than the hair cells themselves^1,2^. After acoustic overexposure, there can be up to 50% loss of these peripheral synapses, even in ears where the noise-induced threshold elevations are only transient in nature. In the aging mouse ear, on average, 50% of the cochlear synapses disappear from surviving inner hair cells (IHCs), despite only ~10% loss of the IHCs themselves^2^. In the aging human ear, there can be as much as 70% loss of cochlear neural connections by 60–70 yrs of age, despite a loss of only ~10% of the IHC population^3^.

Each bipolar cochlear sensory neuron has a cell body in the spiral ganglion that sends a single central axon to the cochlear nucleus and a single peripheral axon to the organ of Corti. Each myelinated peripheral axon gives rise to a single unmyelinated peripheral terminal within the organ of Corti that makes synaptic contact with a single IHC^4^. Given this punctate innervation pattern, each missing IHC synapse corresponds to a silenced cochlear neuron with no spontaneous activity and no response to sound. This type of primary neural degeneration, and the loss of information channels it represents, has little effect on threshold sensitivity until the loss is >80% complete^5,6^, however it likely impairs complex hearing tasks such as word identification in a noisy environment^7,8^ and may also be an important elicitor of tinnitus^9,10^.

After a noise exposure, the peripheral terminals and synaptic contacts of auditory nerve fibers (ANFs) can disappear within hours^11^, and the disconnected peripheral axons can degenerate within a few weeks. However, the cell body and central axon can survive for years in experimental animals^1^ and decades in humans^12^. This degeneration pattern has suggested there might be a therapeutic window within which the problems associated with this type of cochlear synaptopathy could be reversed by treatments designed to elicit neurite outgrowth and synaptogenesis from the surviving spiral ganglion cells. Decades ago, several laboratories showed that, after aminoglycoside-induced destruction of the adult organ of Corti, chronic cochlear perfusion of neurotrophins, e.g. brain-derived neurotrophic factor (BDNF), could elicit neurite extension from surviving spiral ganglion cells^13,14^. These regenerating neurites made their way to the undifferentiated epithelium that had replaced the organ of Corti, and spiraled up and down the cochlea seemingly seeking synaptic targets^14^.

More recently, using transgenic mice, we showed that overexpression of neurotrophin 3 (NT3) in cochlear supporting cells could induce partial synapse rescue and partial restoration of cochlear function after a synaptopathic noise exposure, whereas transgenic overexpression of BDNF was not effective^15^. To test if delivery of exogenous NT3 protein had the same therapeutic utility as transgenic overexpression, we delivered NT3 to the cochlea via the round window membrane 24 hrs after a synaptopathic noise exposure. This approach also regenerated synapses and cochlear function, but only in roughly half the experiments^16^, suggesting that there might be inadequate protein transport through the round window or that the short half-life of the NT3 in cochlear fluids was a problem for developing an effective therapy.

To overcome both of these problems, we turned to a gene-therapy approach using a type of adeno-associated virus (AAV) known to effectively target cochlear hair cells^17,18^. Since NT3 protein injected into the mouse cochlea degrades within 72 hrs post-injection^19^, viral delivery of NT3-expressing vectors could, in comparison, extend the availability of elevated NT3 levels in the cochlea. In a pilot study with reporter-expressing virus, we showed that, by injection of virus suspensions through the posterior semicircular canal, we could transfect IHCs throughout the cochlear spiral in an adult ear without causing any histological damage, cochlear dysfunction, or lingering vestibular problems^18^. Here, we undertook the next step in this progression by assessing the possible therapeutic utility of virally mediated NT3 overexpression by IHCs in rescuing the noise-induced synaptopathy that is reliably created in a mouse model.

## Methods

### Animals and groups

**Figure 1:**
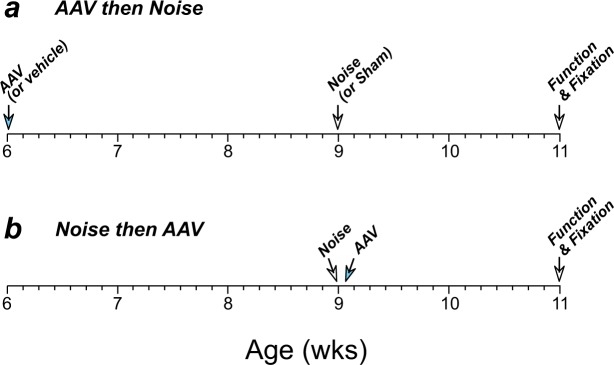
Timelines for the two types of experiments in this study. (**a**) In one protocol, viral suspensions or vehicle were delivered via the PSCC at 6 wks, exposed to noise 3 wks later, allowed to recover for 2 wks, then assessed for cochlear function and/or sacrificed for tissue harvest. (**b**) In the second protocol, animals were exposed to noise at 9 wks, and viral suspensions were delivered via the PSCC within 4.5 hrs post-exposure. Animals recovered for 3 wks, then assessed for cochlear function and sacrificed for tissue harvest.

6-wk, male CBA/CaJ mice were randomly assigned to one of several groups to investigate the effects of virally mediated NT3 overexpression on noise-induced cochlear synaptopathy. As schematized in Fig. 1, some animals were virus-injected 3 wks prior to noise exposure; other age-matched animals were injected within 4–5 hrs after exposure. Controls included virus-injected animals that were sham-exposed, and noise-exposed animals injected with vehicle only. Other animals were injected with the same virus carrying a construct designed to express green fluorescent protein (GFP), to assess the cochlear distribution of viral transfection. Some of these GFP-expressing animals were noise-exposed and others were not. All groups were compared to controls with noise-exposure only (no cochlear injection) as well as animals with neither viral injection nor noise exposure. In all groups, cochlear function was assessed at 11 wks of age, i.e. 2 wks after noise exposure, followed by cochlear fixation and tissue harvest for assessment of hair cell and synaptic damage. All procedures were approved by the IACUC of the Massachusetts Eye and Ear, and were performed in accordance with the “Guide for Care and Use of Laboratory Animals” as prepared by the Committee on Care and Use of Laboratory Animals of the Institute of Laboratory Animal Resources, National Research Council.

### Noise exposure

Mice were placed in individual sections of a wire-mesh cage, rotating within a reverberant enclosure, and exposed to 8–16 kHz noise (fifth-order Butterworth filter) for 2 hrs at 99 dB SPL while unanesthetized and unrestrained. The booth is equipped with a JBL2446H compression driver coupled to an exponential horn and driven by a Crown D75A power amplifier, with a ¼″ Brüel and Kjær condenser microphone to allow precise calibration and monitoring of the sound exposure. The noise level varied by less than 1 dB over the duration of the exposures.

### Viral vectors

**Figure 2:**
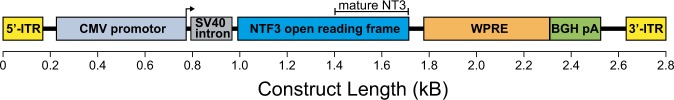
Schematic of the NT3 construct used in the creation of the viral vectors used in the present study. WPRE: woodchuck hepatitis virus posttranscriptional regulatory element; BGH pA: bovine growth hormone poly A; CMV: cytomegalovirus. ITR: inverted terminal repeat.

The gene for GFP or for neurotrophic factor 3 (NT3) were packaged in an AAV2/Anc80L65 adeno-associated virus vector (Anc80), with a cytomegalovirus promoter, a synthetic upstream intron, the woodchuck hepatitis virus posttranscriptional regulatory element, and the bovine growth hormone polyA termination sequence (Fig. 2). The NT3 sequence consisted of the 774-nucleotide coding region for the full pre-pro protein (258 amino acids; UniProtKB accession number P20181) from C57BL/6J mice (Fig. 2). The Gene Transfer Vector Core at the Massachusetts Eye and Ear or the Boston Children’s Hospital Viral Core synthesized the viral vectors. Vector concentrations were between 2.3 × 10^12^ and 3.1 × 10^12^ genome copy/mL, both buffered in 1x PBS + 35 mM NaCl + 0.001% pluronic.

### Posterior semicircular canal injections

The techniques for posterior semicircular canal (PSCC) injections have been described in a prior report^18^. Briefly, mice were anesthetized via intraperitoneal injection of ketamine (100 mg/kg) and xylazine (20 mg/kg), and an incision was made posterior to the pinna. The sternocleidomastoid muscle was trimmed, and the remaining fascia and muscles were retracted to expose the PSCC. A small hole was drilled into the PSCC by pivoting a 26-gauge hypodermic needle on the thin bone covering the canal. After the initial emission of perilymph through the hole subsided, a polyimide tube (039-1, Microlumen) 0.1 mm I.D. was threaded through the opening towards the ampulla. Small pieces of muscle were mixed with VetBond tissue adhesive (3 M) to seal around the tube. The polyimide tube was coupled to a 30-mm polyethylene tube (PE10, I.D. 0.011″, O.D. 0.024″, Becton Dickinson), which itself was coupled to a glass micropipette (#4878, World Precision Instruments). The tubes and micropipette were backfilled with sterile mineral oil to eliminate air pockets, allowing withdrawal and injection of solutions containing virus or vehicle. Injections were made at 100 nL/minute, and allowed to rest for 5 minutes after injection completed before removing the polyimide tube, quickly sealing with PSCC hole with muscle fragments mixed with Vetbond, and closing the incision with nylon sutures.

### Cochlear function testing

In a 32 °C acoustically and electrically shielded room, mice were anesthetized via intraperitoneal injection of ketamine (100 mg/kg) and xylazine (20 mg/kg). A small incision was made on the pinna to provide a clear view of the ear canal and tympanic membrane. The probe tube of a custom acoustic system with miniature drivers (CDMG15008- 03A, CUI) and an electret condenser microphone (FG-23329-PO7, Knowles) was placed within the ear canal, <1 mm outside of the bony ear canal. The acoustic stimuli and physiological responses were digitized by a National Instruments PXI system with 24-bit sound cards running custom LabVIEW software. ABRs were recorded via 30-gauge platinum electrodes, inserted subdermally, adjacent to the pinna incision and at the vertex of the skull, with a ground near the tail. Tone-pip stimuli were presented at half-octave frequency intervals from 5.6–45.2 kHz. At each frequency, a level series was presented, in 5-dB steps, from below threshold to 80 dB SPL, with up to 1024 stimuli averaged per step. Sound-evoked responses were amplified 10,000x through a 0.3–3 kHz bandpass filter. ABR threshold was defined as the lowest stimulus level to generate a waveform that increased in amplitude and decreased in latency as the stimulus level increased. To measure DPOAE threshold, two primary tones were presented at $f2/f1=1.2$ at levels $L1=L2+10\,{\rm{dB}}$, while the distortion product generated at $2f1\,-\,f2$ was recorded. The f2 stimuli were presented at 5.6 kHz–45.2 kHz at half-octave intervals from 10–80 dB SPL. DPOAE threshold was defined as the interpolated value of f2 intensity required to generate a 0 dB SPL DPOAE. ABR and DPOAE thresholds are expressed as threshold shifts, i.e. observed threshold minus the mean control threshold at the same test frequency.

### Histological tissue processing and immunostaining

Prior to tissue harvest, the animals were transcardially perfused with 4% paraformaldehyde in phosphate buffer. Cochleas were flushed with fixative through the scalae and post-fixed for 2 hrs, decalcified in EDTA, and dissected into half turns. Tissue was permeabilized by freezing on dry ice in 30% sucrose, blocked for 1 hr at 22 °C in PBS with 0.03% Triton X + 5% normal horse serum, and washed in PBS. Tissue was then incubated overnight at 37 °C in the following primary antibodies: (1) mouse isotype IgG1 anti-C-terminal binding protein 2 (CtBP2, 1:200, BD Transduction Laboratories #612044), (2) mouse isotype IgG2 anti-glutamate receptor 2 (GluA2, 1:2000, Millipore #MAB397), (3) rabbit anti-myosin VIIa (Myo7a, 1:200, Proteus BioSciences #25–6790), and mouse anti-neurofilament H (NFH, 1:1000, Millipore #AB5539). After rinsing, tissue was incubated twice for 1 hr at 37 °C in the following secondary antibodies: (1) goat anti-mouse IgG1 Alexa Fluor 568 conjugate (1:1000, Thermo Fisher #A-21124), (2) goat anti-mouse IgG2a Alexa Fluor 488 conjugate (1:1000, Thermo Fisher #A-21131), (3) goat anti-chicken Alexa Fluor 647 (1:200, Thermo Fisher #A-21449), and (4) goat anti-rabbit PacificBlue (1:200, Thermo Fisher #P-10994).

### Hair cell and synaptic loss

Dissected cochlear pieces were imaged with a low-power objective, using the signal from the myosin VIIa channel. A cochlea length and frequency map was generated using a custom ImageJ plugin. Cochlear frequency was calculated using the formula, $F(kHz)=({10}^{d\times 0.92}-0.680)\times 9.8$, where d is the fractional distance from the apex^20,21^. Hair cell loss was analyzed as previously described^22^ by dividing the cochlear spiral into 20 equal-length bins and calculating the fractional loss of HCs observed within each bin. Cochlear synapse counts were derived from confocal z-stacks from two adjacent 80-μm fields per frequency place, imaged at half-octave intervals from 8–64 kHz using a Leica TCS SP8 confocal microscope with a 63x glycerol-immersion objective (N.A. = 1.3) and 2.4x digital zoom. A synapse was defined as a CtBP2-stained punctum that is immediately adjacent to GluA2-stained punctum. Similar imaging and analysis parameters were used in all subjects and at all frequencies. Synaptic losses were calculated as the fractional difference of the number of synapses per IHC in experimental animals compared to untreated control ears at each frequency place.

### RNA expression

To quantify NT3 expression levels after Anc80-NT3 injection, cochleas were collected at several post-injection time points after delivery of 250 or 1000 nL Anc80-NT3, or from uninjected control animals. Inner ears were removed and placed immediately in RNALater (Thermo Fisher #AM7020), where cochleas were separated from vestibular end organs and any contaminating tissue before being homogenized. Relative changes in NT3 RNA expression were calculated by qRT-PCR using the ΔΔCt method, normalized to the housekeeping gene, ribosomal protein L19. RNA was reverse transcribed using an iScript cDNA Synthesis Kit (Bio-Rad) in a 20-µl reaction. The reverse transcription was performed at 42 °C for 45 min followed by 85 °C for 5 min. qPCRs were run in a CFX96 machine and QuantStudio 5 Real-Time PCR machine using an iTaq Universal SYBR Green Supermix (Bio-Rad). The following PCR primers were used: Ntf3 forward: GCCCCCTCCCTTATACCTAATG, Ntf3 reverse: CATAGCGTTTCCTCCGTGGT; Rpl19 forward: ACCTGGATGAGAAGGATGAG, Rpl19 reverse: ACCTTCAGGTACAGGCTGTG.

### Statistics methodology

All statistical comparisons were made using Graphpad Prism v8 software. ANOVAs were adjusted for repeated measures using a mixed-model 2-way ANOVA that accounts for missing values while, in the absence of missing values, calculates the same p values as normal 2-way repeated-measures ANOVAs. Any *post hoc* multiple comparisons were calculated using the Holm-Sidak multiple comparisons method. Pairwise comparisons were made using a nonparametric, 2-tailed Mann-Whitney test.

## Results

### Virally mediated NT3 overexpression in the cochlea

**Figure 3:**
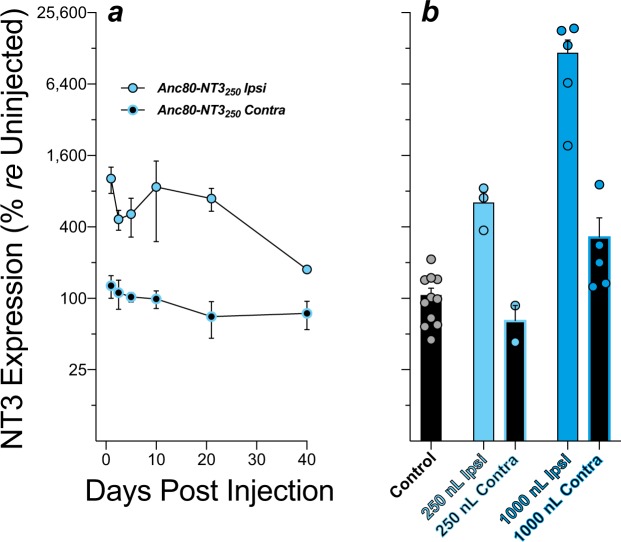
Virally mediated increases in cochlear NT3 expression, as assessed by qRT-PCR. (**a**) NT3 mRNA levels in injected and contralateral cochleas at post-injection days 1, 2.5, 5, 10, 21 and 40, normalized to mean levels in the contralateral ears. (**b**) To assess contralateral spread of the virus, NT3 mRNA levels were measured in both ears at 21 days post-injection and normalized to levels in uninjected controls. Histograms show means and SEMs; individual cases are shown by the colored circles in **b**.

Using a GFP reporter, we have previously shown that Anc80 virus injected through the PSCC in mouse can efficiently transfect IHCs through the entire length of the cochlea, when evaluated 2 wks after injection. However, it was not known how quickly this overexpression occurs, how dramatically expression can be enhanced, and for how long the overexpression can be maintained^18^. To determine this, we analyzed cochlear expression of NT3 via qRT-PCR at 1, 2.5, 5, 10, 21, and 40 days after a 250 nL PSCC injection of Anc80-NT3. As shown in Fig. 3a, NT3 is overexpressed by >8-fold *re* the contralateral ear at 24 hrs post-injection, and overexpression remains 4- to 10-fold higher than the contralateral ear for at least 21 days.

To clarify whether the virus was spreading to the contralateral ear, cochleas from uninjected animals were compared to both ipsilateral and contralateral ears of another set of injected animals, 21 days after PSCC delivery of either 250- or 1000-nL Anc80-NT3 (Fig. 3b). Relative to uninjected controls, NT3 expression after 250-nL injection was ~6-fold higher (p = 0.0055), and there was no evidence of leakage to the contralateral ear. Injections of 1000-nL Anc80-NT3 increased the NT3 expression by ~100-fold ipsilaterally and significantly increased contralateral expression as well, *re* uninjected controls (p = 0.0005 and p = 0.038 respectively).

### Effects of NT3 overexpression on noise-induced synaptopathy and hair cell loss

Moderate noise exposure can cause the permanent loss of cochlear afferent synapses on IHCs, even if post-exposure thresholds return to normal and there is no loss of hair cells^1,23^. Previous studies have shown that both transgenically driven NT3 overexpression in cochlear supporting cells and post-exposure delivery of NT3 protein at the round window can regenerate the synapses lost after neuropathic noise^15,16^. We tested whether similar protection or regeneration could be achieved in a gene-therapy model, by overexpressing NT3 in cochlear hair cells with an Anc80 AAV^17,18,24^, delivered either before or after the noise.

**Figure 4:**
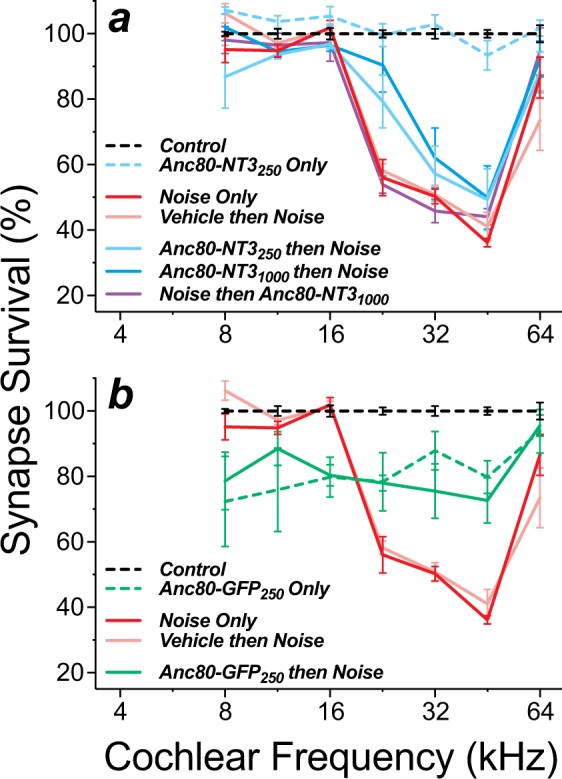
NT3 overexpression reduced noise-induced synaptopathy (**a**), while GFP overexpression increased synaptopathy with or without noise (**b**). (**a**) Summary of synaptic survival for AAV-NT3 groups and relevant controls. Group sizes were as follows: *Controls* (n = 4), *Noise Only* (n = 7), *Vehicle then Noise* (n = 4), *Anc80-NT3*_250_ *Only* (n = 4), *Anc80-NT3*_250_ *then Noise* (n = 7), *Anc80-NT3*_1000_ *then Noise* (n = 7) *and Noise then Anc80-NT3*_1000_ (n = 5). (**b**) Summary of synapse survival for AAV-GFP groups and relevant controls. *Control*, *Noise Only* and *Vehicle then Noise* groups are the same as in Panel a. Group sizes for the GFP groups were as follows: *Anc80-GFP*_250_ *Only* (n = 4), and *Anc80-GFP*_250_ *then Noise* (n = 5). Means (±SEMs) are shown in all plots. All data are normalized to mean values in unexposed controls.

We injected Anc80-NT3 either 3 wks before, or 4–5 hrs following, a 2-hr 99-dB exposure to octave-band noise exposure that, in the absence of therapeutic intervention, reliably causes significant cochlear synaptopathy in mice (Fig. 4a; *Noise Only*)^1,23^. In the absence of noise exposure, virus-mediated NT3 overexpression did not cause synaptopathy relative to unexposed controls (Fig. 4a; *Anc80-NT3*_250_ *Only* vs*. Control*). Ears pre-treated with either 250- or 1000-nL suspensions of virus (Fig. 4; *Anc80-NT3 then Noise*) displayed a frequency-dependent reduction in synaptopathy *re* noise-exposed untreated and noise-exposed vehicle-treated ears (p < 0.05 by 2-way ANOVA), particularly at the 22.6-kHz region of the cochlea (250-nL p < 0.05, 1000 nL p < 0.0001). Post-exposure Anc80-NT3 treatment showed no effects, with such ears displaying synaptic pathology similar to untreated noise-exposed ears (Fig. 4a: *Noise then Anc80-NT3* vs. *Noise Only*).

**Figure 5:**
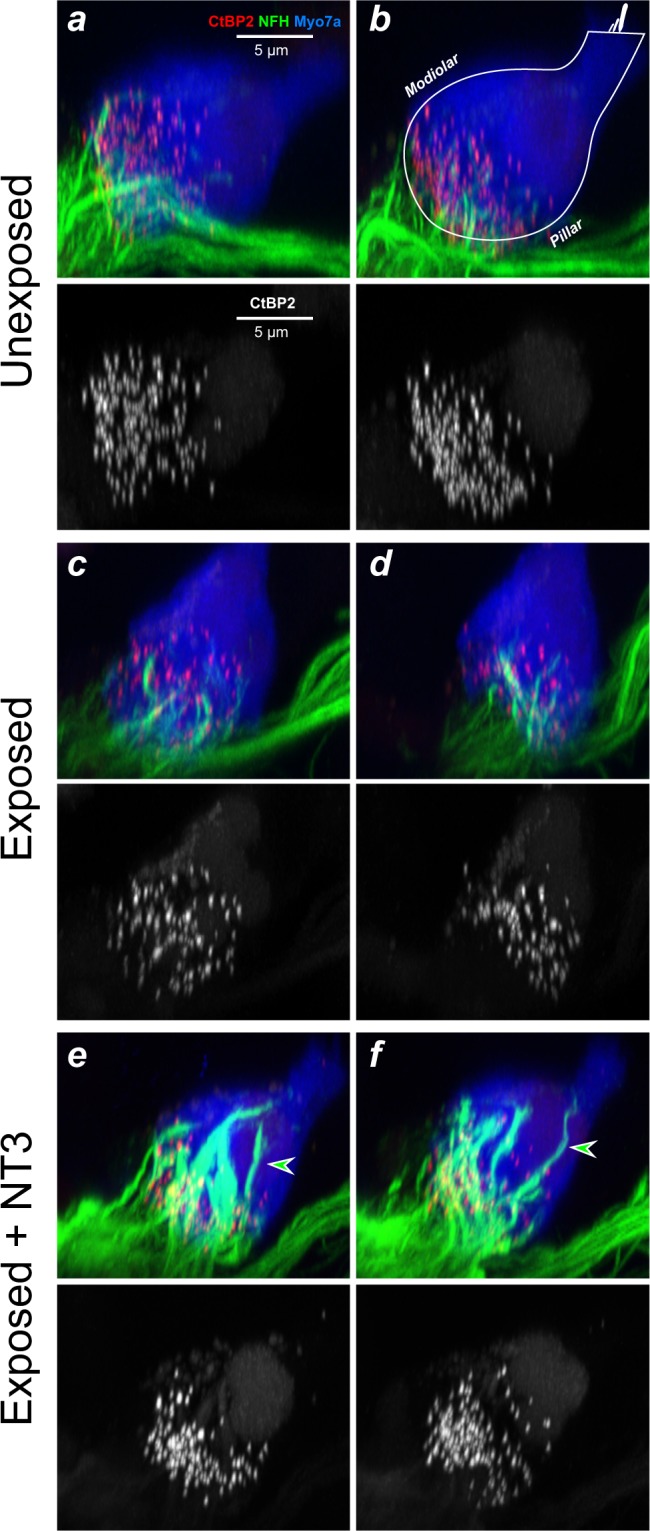
NT3 overexpression reduces synaptopathy and maintains the ribbon-size gradient, but changes location and immunostaining of surviving ANF terminals. Images here are from the 32-kHz region of three animals, from the groups indicated. Each is a re-projection into the yz plane of a confocal z-stack spanning ~10 IHCs (77 μm) along the cochlear spiral. These yz maximum projections mimic thick cross-sections through the organ of Corti. Each stack is shown as both a 3-color image showing immunostaining of IHCs (Myo7a, blue), ribbons (CtBP2, red), and neurons (NFH, green), and a greyscale image to show just the synaptic ribbons. The white outline in panel **b** illustrates the orientation of the IHC and its stereocilia bundle in these yz projections (OHCs are to the right). Arrowheads indicate terminals that project farther from the synaptic pole, and appear richer in neurofilaments, than normal. The scale bar in **a** applies to all panels.

In the normal cochlea, the synaptic ribbons show a modiolar-pillar size gradient, which reflects the modiolar-pillar segregation of ANFs with different spontaneous rates and threshold sensitivity^25–27^. Such a gradient is visible in the confocal images from unexposed ears (Fig. 5a,b). As reported previously^28^, the gradient is disrupted in cochlear regions with significant synaptopathy (e.g. compare Fig. 5a,c). Importantly, in the cochlear regions where the NT3 was effective in minimizing synaptopathy, the ribbon-size gradient appears to also be maintained (Fig. 5e,f).

**Figure 6:**
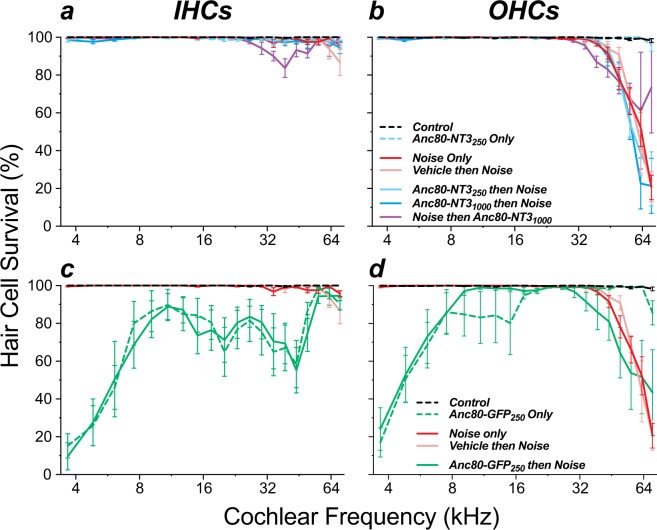
NT3 overexpression did not affect noise-induced hair cell loss (**a,b**), while GFP overexpression caused IHC loss, with or without noise exposure (**c,d**). Means (±SEMs) are shown. Group sizes were as follows: *Controls* (n = 6), *Noise Only* (n = 7), *Vehicle then Noise* (n = 4), *Anc80-NT3*_250_ *Only* (n = 4), *Anc80-NT3*_250_ *then Noise* (n = 7), *Anc80-NT3*_1000_ *then Noise* (n = 7), *Noise then Anc80-NT3*_1000_ (n = 5), *Anc80-GFP*_250_ *Only* (n = 5), and *Anc80-GFP*_250_ *then Noise* (n = 5). Survival values are absolute, i.e. not normalized.

Prior studies have suggested that exogenous neurotrophic factors can reduce the noise-induced loss of OHCs^16,29,30^. Here, NT3 overexpression had minimal effects on hair cell survival. Although our noise exposure was designed to cause synaptopathy with minimal HC death, all noise-exposed groups showed a tonotopically inappropriate OHC loss in the extreme base (Fig. 6b,d) that is very common after this type of 1–4 hr exposure^31,32^. IHC death rarely exceeded 5% regardless of exposure or NT3 virus dose (Fig. 6a). Some OHC protection was observed at the basalmost cochlear location, comparing ears treated post-exposure to exposed ears receiving no treatment (p < 0.0001). OHC loss was statistically indistinguishable, however, between noise-exposed cohorts that were untreated, vehicle treated, or Anc80-NT3 injected *before* exposure (p = 0.77 by 2-way ANOVA).

Other studies have suggested that virally mediated NT3 overexpression can lead to exuberant overgrowth of cochlear nerve terminals in the organ of Corti^33^. To address this issue, we included anti-neurofilament immunostaining in all the cochlear preparations. This immunostaining labels the unmyelinated terminals of ANFs in the synaptic region of the IHCs. As shown in the confocal maximum projections of Fig. 5, there were clear alterations in the morphology of ANF terminals in the NT3-overexpressing ears: the neurofilament staining was much stronger, and the ANF terminals extended farther from the basolateral pole of the IHC, well beyond the cloud of pre-synaptic ribbons (arrowheads in Fig. 5e,f). However, ANF terminals did not appear to project to any other target outside of the IHC area. Examining this type of re-projected confocal image from all 16-, 22-, and 32-kHz z-stacks in the present study, an observer blinded to treatment group sorted all Anc80-NT3-treated, vehicle-treated, and control ears into categories based on whether or not the morphology displayed abnormal afferent outgrowth. This observer correctly identified 95% of all Anc80-NT3-treated ears and 0% of control ears as displaying increased terminal outgrowth. This Anc80-NT3-induced neurite extension was observed more often at higher frequencies, with 52.9%, 81.3%, and 94.7% identified at 16 kHz, 22 kHz, and 32 kHz respectively.

### Effects of GFP overexpression on noise-induced synaptopathy and hair cell loss

A prior study of mice examined 2 wks after cochlear injection of Anc80-GFP saw no histopathology or cochlear dysfunction from the GFP overexpression^18^. Here, to match the time course of Anc80-NT3 pretreated ears (Fig. 1a), Anc80-GFP ears were examined 5 wks after virus injection, rather than 2 wks. At this longer post-injection survival, the GFP-expressing ears showed moderate synaptopathy throughout the cochlea, except at the most basal tip (Fig. 4b). The synaptic loss was nearly identical regardless of noise exposure (Fig. 4b; *Anc80-GFP then Noise* vs *Anc80-GFP Only*; p = 0.98 by 2-way ANOVA). Paradoxically, in the basal half of the cochlea, the noise-exposed GFP overexpressers showed less synaptopathy than untreated noise-exposed ears *(Noise Only* p < 0.01 at 22–45 kHz by Holm-Sidak’s multiple comparisons test).

The prolonged (5 wk) GFP overexpression also resulted in significant, frequency-dependent IHC and OHC death (Fig. 6c,d; p < 0.0001 by 2-way ANOVA). IHC loss was significantly increased throughout the entire cochlea, except the extreme base, and increased from cochlear middle to cochlear apex. As seen for synaptopathy, the degree of hair cell death was unaffected by noise exposure (Fig. 6c), perhaps because the GFP overexpression had jeopardized mechanoelectric transduction in IHCs, thereby protecting them from metabolic overload (see below). OHC loss also increased dramatically from the cochlear middle to the apex, while, in the basal half, the loss matched that seen in the appropriate control ear (Fig. 6d: compare *Anc80-GFP then Noise* to *Noise Only* or compare *Anc80-GFP Only* to *Control*).

### Effects of GFP or NT3 overexpression on cochlear function and noise-induced threshold shifts

**Figure 7:**
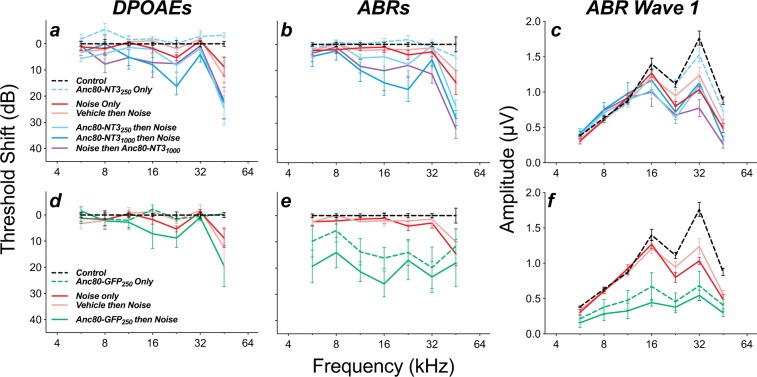
NT3 overexpression slightly increased noise-induced threshold shift in both DPOAEs (**a**) and ABRs (**b**), with a concomitant reduction in wave 1 amplitudes (**c**). GFP overexpression similarly increased noise-induced DPOAE thresholds shifts (**d**), while greatly increasing ABR thresholds (**e**) and reducing ABR wave 1 amplitudes (**f**) with or without noise exposure. Groups and group sizes were as follows: *Controls* (n = 15), *Noise Only* (n = 10), *Vehicle then Noise* (n = 5), *Anc80-NT3*_250_ *Only* (n = 6), *Anc80-NT3*_250_ *then Noise* (n = 7), *Anc80-NT3*_1000_ *then Noise* (n = 8) *and Noise then Anc80-NT3*_1000_ (n = 5). Group sizes for the GFP groups were as follows: *Anc80-GFP*_250_ *Only* (n = 7), and *Anc80-GFP*_250_ *then Noise* (n = 5). Wave 1 amplitude is extracted from each case at each frequency by averaging results from stimulus levels of 60, 70, and 80 dB SPL. Means (±SEMs) are shown in all plots. All data are normalized to mean values in unexposed controls.

Anc80-NT3 injections without noise exposure did not significantly alter ABR thresholds, when measured 5 wks after injection (Fig. 7a,b; p = 0.622) and slightly improved DPOAE thresholds (p = 0.0326 by 2-way ANOVA). Despite reducing noise-induced synaptopathy, with little effect on HC survival (Figs 4 and 6), Anc80-NT3 injections increased the susceptibility of treated ears to noise-induced permanent threshold shift (PTS; Fig. 7a,b). Animals pretreated with 1000 nL Anc80-NT3 showed a frequency-dependent increase in PTS, whether measured by ABR (Fig. 7b; p = 0.030) or DPOAE (Fig. 7a; p = 0.008). Animals pretreated with 250 nL Anc80-NT3 displayed increased DPOAE threshold shifts (Fig. 7a; p = 0.043), but no significant increase ABR PTS (Fig. 7b; p = 0.19). Animals treated post-exposure with 1000 nL Anc80-NT3 displayed a frequency-dependent increase in ABR PTS (p = 0.010), and a nearly significant increase in DPOAE PTS (p = 0.053). The exacerbation of noise-induced DPOAE (and ABR) shifts in NT3-overexpressing ears suggests increased OHC vulnerability. Since IHC synaptopathy does not affect thresholds (either ABR or DPOAE)^1^, the synaptic rescue elicited by NT3 overexpression cannot counterbalance these shifts.

The widespread IHC distress after prolonged GFP expression, suggested by the cell death patterns (Fig. 6c), is also reflected in ABR thresholds (Fig. 7e). Significant differences in ABR threshold shifts were observed in unexposed GFP-expressing ears *re* unexposed untreated controls, and in exposed GFP-expressing ears *re* exposed untreated ears (p < 0.0001 and p = 0.0002, respectively, by 2-way ANOVA). In contrast, despite considerable loss of OHCs in the apical cochlea (Fig. 6d), unexposed GFP-expressing ears had DPOAE thresholds similar to controls (Fig. 7d; p = 0.545 by 2-way ANOVA), and GFP-expressing ears exposed to noise were similar to noise-exposed controls (Fig. 7d; p = 0.167). The fact that ABR shifts are worse than DPOAE shifts suggests that IHC damage from GFP overexpression is worse than OHC damage. This, in turn, is consistent with the difference in intensity of GFP expression and degree of cell death (Fig. 6c,d) between the two cell types.

For cases in which cochlear thresholds remain at, or return to, normal values, the suprathreshold amplitudes of the ABR wave 1, the summed activity of the auditory nerve, are proportional to numbers of surviving synapses^1^. Here, the cochlear thresholds at the end of the experiment were not normal except in the unexposed, Anc80-NT3 injected ears (Fig. 7a,b), thus the suprathreshold wave 1 amplitudes (Fig. 7c,f) are not easy to interpret and are not useful in determining whether the rescued synapses were functional. Nevertheless, the similarity in wave 1 amplitudes among all the noise-exposed groups (Fig. 7c), except those treated with Anc80-GFP (Fig. 7f), suggests that the Anc80-NT3 treatment has not caused any unexpected suprathreshold response decrements beyond those attributable to the threshold elevations.

## Discussion

### Presence vs. absence of therapeutic effects

Here, we show that virally mediated NT3 overexpression in IHCs can significantly reduce the degree of noise-induced synaptopathy, if initiated before the noise exposure (Fig. 4a). The protective effect decreased from the apical to the basal end of the lesion: the improvement in synaptic survival observed was 34%, 12%, 14% and 5.7% at 22.6 kHz, 32.0 kHz, 45.2 kHz and 64.0 kHz respectively. This gradient of rescue is consistent with the overall gradient of forced overexpression that we infer from a variety of related observations following AAV injection in the PSCC. First, in a prior study from our lab using exactly the same techniques^18^, qualitative observation suggested that, although virtually all IHCs were transfected by the same GFP-expressing virus used here, the reporter signal was progressively weaker from apex to base (Fig. 1a in Suzuki *et al*.^18^). In the OHC area, where only a fraction of cells showed GFP signal, quantitative analysis from confocal images showed that the number of transfected cells decreased monotonically from apex to base. Second, in the present study we observed a decreasing apex-to-base gradient of IHC and OHC death 5 wks after injection of the GFP-expressing virus (Fig. 6), consistent with a toxic effect of GFP overexpression (see below) and a decreasing gradient of this overexpression from apex to base. Such a gradient likely arises because the diffusion path for virus particles from scala tympani (through the basilar membrane) to hair cells is more direct than that from scala vestibuli. Thus, the most consistent transfection starts in the apex, after the injected bolus diffuses up scala vestibuli from the PSCC injection site, and travels through the helicotrema to begin accumulating in scala tympani in an apex to base gradient.

Here, we also showed that rescue from noise-induced synaptopathy could not be achieved when the virus was injected after exposure, even with a trauma-treatment interval of only 4–5 hrs (Fig. 4a). This was surprising, given our prior experiments with transgenic NT3 expression immediately post-exposure, and with round-window application of NT3 protein in a slow-release gel a full 24 hrs post-exposure, both of which showed significant rescue of noise-induced synaptopathy^15,16^. Although the round-window delivery route presumably gives more rapid access to the synaptopathic region (in the basal turn), our mRNA expression data show that, after PSCC injection, whole-cochlear NT3 expression levels have already risen to peak levels by 24 hrs post-injection (Fig. 3a). Given this rapid increase in transcription of NT3 mRNA, it seems unlikely that NT3 protein translation would lag more than a few hours behind.

A number of explanations can be considered for this failure of post-trauma rescue by NT3 gene therapy. One important difference between the two approaches is that, with round-window protein delivery, the neurotrophin is distributed via the cochlear fluids and thus will theoretically reach any tissues bathed in perilymph, including both the spiral ganglion cells and the organ of Corti. By contrast, in the IHC overexpression strategy, since Anc80-NT3 does not effectively transduce neurons or supporting cells^17,18^, the protein is likely more restricted to the region directly around the peripheral synaptic terminals. Since these terminals appear to explode and retract during the two-hour exposures^11^, this may remove the relevant Trk receptors from close proximity to the NT3 source in this IHC-based gene-therapy approach. If, in a post-trauma treatment protocol, it is important to engage neurotrophin receptors farther from the IHCs, e.g. on the cell bodies or peripheral axons of the spiral ganglion neurons, a gene-therapy approach that elicits overexpression in the IHCs might be less effective than a protein on the round-window approach or a transgenic approach, in which the supporting cells, rather than the IHCs, are overexpressing the NT3. In addition, substantially higher levels of NT3 transcripts (10-fold vs 2-fold) were produced by the AAV vector here, relative to the conditional transgenic NT3^15^. While the transcripts in both approaches encoded the mouse pre-pro NT3 protein, the presumed higher levels of immature NT3 produced following AAV delivery may have resulted in suboptimal processing, secretion, or activity of the mature, active neurotrophin^34^.

### Enhanced protection vs. enhanced recovery

Given the experimental design, we do not know whether the reduction in final synaptopathy in the pre-trauma gene-delivery experiments reflects a decrease in initial damage, an increase in post-exposure recovery/regeneration, or both. The obvious changes in the staining properties of the unmyelinated ANF terminals, and of their morphological arrangement along the basolateral surfaces of the IHCs (Fig. 5), demonstrate that an active NT3 must be reaching the Trk receptors on these terminals and eliciting neurite extension after noise exposure. Thus, the gene therapy delivered here may well be eliciting post-exposure regeneration of damaged terminals.

In prior work, using round-window delivery of NT3 protein, we demonstrated the functional recovery of regenerated synapses in treated animals by measuring the suprathreshold amplitude of ABR wave 1 in response to tone pips in the affected cochlear frequency regions^16^: ears with the highest fractional synaptic recovery also showed the highest fractional recovery of the wave 1 amplitudes. In this study, we cannot interpret the ABR suprathreshold data, because, here, the noise-exposed, treated animals (*Anc80-NT3 then Noise*) showed significantly larger permanent threshold shifts than the exposed untreated or exposed vehicle-treated animals (*Noise only* & *Vehicle then Noise*; Fig. 7): this enhanced noise vulnerability of treated animals was not observed in our prior study of round-window NT3 delivery.

Increased noise vulnerability was observed here in the post-exposure thresholds measured via either ABRs and DPOAEs (Fig. 7a,b, respectively). The DPOAE effects in exposed-treated ears suggest that outer hair cells, although they remain functional in unstressed ears even for fully 5 wks after forced NT3 overexpression, are revealed to be abnormal by their reduced resistance to the effects of high-level acoustic overstimulation. The ABR threshold shifts in the same animals may simply reflect the downstream effect of the OHC dysfunction, however, they may also reflect increased vulnerability to stress in the IHCs themselves. This, in turn, raises the possibility that the reduction of synaptic loss in the exposed-treated ears arises because, during the exposure, the accumulating hair cell damage reduces glutamate release at the IHC/ANF synapse, and thereby reduces the amount of synaptopathy observed *re* the untreated-exposed animals. It may be significant in this regard that the region of maximum ultimate PTS (22.6 kHz; Fig. 7a,b) is the same as the cochlear region of maximum synaptic “protection” (22.6 kHz; Fig. 4a).

A similar line of thinking applies to the surprising observation that the forced GFP-overexpression reduces synaptic counts even in unexposed ears (Fig. 4b), but then renders these ears completely resistant to further noise-induced synaptopathy. We hypothesize that the cellular demands of protein overproduction, or the accumulation of intracellular GFP *per se*, compromises IHC function and reduces its ability to transduce mechanical vibration into auditory-nerve excitation. This putative IHC dysfunction could arise anywhere along the transduction cascade from mechano-electric transduction to voltage-activated vesicle release. The idea that acoustically driven glutamate release from the IHCs is reduced in these ears is further supported by the observations that the ABR thresholds in unexposed GFP-expressing ears are much more elevated than the DPOAE thresholds, and that the GFP-expressing ears display the lowest wave-1 amplitudes of any groups in this study (Fig. 7). Others have shown that elimination of synaptic transmission at the IHC/ANF synapse eliminates noise-induced synaptopathy even in the absence of OHC dysfunction^35^.

### Toxicity and alternate approaches

While this and prior studies in our lab have found Anc80-driven GFP expression is well tolerated in cochlear HCs at 2 wks post-injection (data not shown & Suzuki *et al*.^18^), prolonged GFP expression resulted in HC death and compromised cochlear function (Figs 6 and 7). The degeneration patterns observed here mirror the GFP expression patterns reported in our prior study: i.e. IHC expression throughout the cochlea, but qualitatively stronger in the apex than the base, and OHC expression only detectable in the apical half of the cochlea^18^. Similar toxicity has been shown in other systems, where GFP expression induced immunogenic and apoptotic death^36,37^. GFP expression causes dose-dependent toxicity^38^ and is more toxic than similar fluorescent transgenes delivered with identical vehicles^37^. Relatedly, while we observe HC death is induced by GFP expression, this toxicity is not observed with vehicle injections, or injections of the NT3 transgene (Fig. 6). Absent noise exposure, unlike Anc80-GFP injections, vehicle and Anc80-NT3 injections do not induce threshold shifts, however ears overexpressing the NT3 transgene do show greater noise-induced PTS compared to noise-exposed vehicle-treated and untreated ears (Fig. 7). This suggests that while NT3 expression in HCs is tolerated better than GFP, the added burden of transgene overexpression may leave fragile HCs more susceptible to noise-induced stress.

A previous study of AAV-NT3 transfection of IHCs prior to noise exposure showed partial rescue of synapses without an increase in permanent threshold shift, but also without the expected rescue of cochlear function as seen in suprathreshold ABR amplitudes^39^: the prior study used a different viral serotype (AAV8), a different species (guinea pig), a different delivery route (cochleostomy), a different treatment-trauma interval (1 wk) and a different trauma-sacrifice interval (2 wks). Unfortunately, the degree of NT3 overexpression was not assayed, so it’s difficult to speculate as to the reason(s) for the differences in apparent hair cell vulnerability.

AAVs are an increasingly popular vector for gene therapy due in part to their transduction efficiency and low toxicity^40^, however, while few *in vivo* studies have investigated recombinant AAV toxicity without the potential confound of transgene toxicity, some reports have indicated that localized high doses of certain AAV vectors could have some inherent toxicity^41,42^. Anc80 was chosen as an ideal serotype in this study to target NT3 overexpression in HCs. While Anc80-delivered NT3 in HCs did effectively protect against noise-induced synaptopathy, given the negative effects we observe with transgene expression in HCs in this study, a better route may be to transduce NT3 in the supporting cells adjacent to HCs, providing the therapeutic benefit of increased extracellular NT3 around IHC synapses without stressing the fragile HCs themselves. In addition, these results suggest that determining the side-effects viral vectors have on cochlear morphology and function, at different target levels of forced overexpression and in both unexposed and noise-exposed conditions, are essential factors to consider in future cochlear gene therapy investigations.

## Data Availability

The datasets generated and analyzed in this study are available from the corresponding author on request.

## References

[1] Kujawa SG, Liberman MC (2009). Adding insult to injury: cochlear nerve degeneration after “temporary” noise-induced hearing loss. J Neurosci.

[2] Sergeyenko Y, Lall K, Liberman MC, Kujawa SG (2013). Age-related cochlear synaptopathy: an early-onset contributor to auditory functional decline. J Neurosci.

[3] Wu PZ (2019). Primary Neural Degeneration in the Human Cochlea: Evidence for Hidden Hearing Loss in the Aging Ear. Neuroscience.

[4] Liberman MC (1982). Single-neuron labeling in the cat auditory nerve. Science.

[5] Lobarinas Edward, Salvi Richard, Ding Dalian (2013). Insensitivity of the audiogram to carboplatin induced inner hair cell loss in chinchillas. Hearing Research.

[6] Schuknecht HF, Woellner RC (1955). An experimental and clinical study of deafness from lesions of the cochlear nerve. J Laryngol Otol.

[7] Liberman MC, Epstein MJ, Cleveland SS, Wang H, Maison SF (2016). Toward a Differential Diagnosis of Hidden Hearing Loss in Humans. PLoS One.

[8] Lobarinas E, Salvi R, Ding D (2016). Selective Inner Hair Cell Dysfunction in Chinchillas Impairs Hearing-in-Noise in the Absence of Outer Hair Cell Loss. J Assoc Res Otolaryngol.

[9] Bauer CA, Brozoski TJ, Myers K (2007). Primary afferent dendrite degeneration as a cause of tinnitus. J Neurosci Res.

[10] Hickox AE, Liberman MC (2014). Is noise-induced cochlear neuropathy key to the generation of hyperacusis or tinnitus?. J Neurophysiol.

[11] Liberman LD, Suzuki J, Liberman MC (2015). Dynamics of cochlear synaptopathy after acoustic overexposure. J Assoc Res Otolaryngol.

[12] Liu W (2015). The pre- and post-somatic segments of the human type I spiral ganglion neurons–structural and functional considerations related to cochlear implantation. Neuroscience.

[13] Miller JM (1997). Neurotrophins can enhance spiral ganglion cell survival after inner hair cell loss. Int J Dev Neurosci.

[14] Wise AK, Richardson R, Hardman J, Clark G, O’Leary S (2005). Resprouting and survival of guinea pig cochlear neurons in response to the administration of the neurotrophins brain-derived neurotrophic factor and neurotrophin-3. J Comp Neurol.

[15] Wan, G., Gómez-Casati, M. E., Gigliello, A. R., Liberman, M. C. & Corfas, G. Neurotrophin-3 regulates ribbon synapse density in the cochlea and induces synapse regeneration after acoustic trauma. *Elife* **3** (2014). 10.7554/eLife.03564 PMC4227045 25329343

[16] Suzuki J, Corfas G, Liberman MC (2016). Round-window delivery of neurotrophin 3 regenerates cochlear synapses after acoustic overexposure. Sci Rep.

[17] Landegger LD (2017). A synthetic AAV vector enables safe and efficient gene transfer to the mammalian inner ear. Nat Biotechnol.

[18] Suzuki J, Hashimoto K, Xiao R, Vandenberghe LH, Liberman MC (2017). Cochlear gene therapy with ancestral AAV in adult mice: complete transduction of inner hair cells without cochlear dysfunction. Sci Rep.

[19] Hu, Q. Y. *et al*. In *42nd Annual MidWinter Meeting*. 449 (Association for Research in Otolaryngology, 2019). 10.1007/s10162-024-00930-9 PMC10933225 38308691

[20] Taberner AM, Liberman MC (2005). Response properties of single auditory nerve fibers in the mouse. J. Neurophysiol..

[21] Muller M, von Hunerbein K, Hoidis S, Smolders JW (2005). A physiological place-frequency map of the cochlea in the CBA/J mouse. Hear Res.

[22] Hickman TT, Smalt C, Bobrow J, Quatieri T, Liberman MC (2018). Blast-induced cochlear synaptopathy in chinchillas. Sci Rep.

[23] Liberman MC, Kujawa SG (2017). Cochlear synaptopathy in acquired sensorineural hearing loss: Manifestations and mechanisms. Hear Res.

[24] Zinn E (2015). In Silico Reconstruction of the Viral Evolutionary Lineage Yields a Potent Gene Therapy Vector. Cell Rep.

[25] Liberman LD, Wang H, Liberman MC (2011). Opposing gradients of ribbon size and AMPA receptor expression underlie sensitivity differences among cochlear-nerve/hair-cell synapses. J Neurosci.

[26] Hickman TT, Liberman MC, Jacob MH (2015). Adenomatous Polyposis Coli Protein Deletion in Efferent Olivocochlear Neurons Perturbs Afferent Synaptic Maturation and Reduces the Dynamic Range of Hearing. J Neurosci.

[27] Liberman LD, Liberman MC (2016). Postnatal maturation of auditory-nerve heterogeneity, as seen in spatial gradients of synapse morphology in the inner hair cell area. Hear Res.

[28] Furman AC, Kujawa SG, Liberman MC (2013). Noise-induced cochlear neuropathy is selective for fibers with low spontaneous rates. J. Neurophysiol..

[29] Shoji F (2000). Differential protective effects of neurotrophins in the attenuation of noise-induced hair cell loss. Hear Res.

[30] Ruan RS, Leong SK, Mark I, Yeoh KH (1999). Effects of BDNF and NT-3 on hair cell survival in guinea pig cochlea damaged by kanamycin treatment. Neuroreport.

[31] Fried MP, Dudek SE, Bohne BA (1976). Basal turn cochlear lesions following exposure to low-frequency noise. Transactions of the American Academy of Ophthalmology and Otolaryngology.

[32] Liberman MC, Kiang NY (1978). Acoustic trauma in cats. Cochlear pathology and auditory-nerve activity. Acta oto-laryngologica.

[33] Lee MY (2016). Viral-mediated Ntf3 overexpression disrupts innervation and hearing in nondeafened guinea pig cochleae. Mol Ther Methods Clin Dev.

[34] Farhadi HF (2000). Neurotrophin-3 sorts to the constitutive secretory pathway of hippocampal neurons and is diverted to the regulated secretory pathway by coexpression with brain-derived neurotrophic factor. J Neurosci.

[35] Kim KX (2019). Vesicular Glutamatergic Transmission in Noise-Induced Loss and Repair of Cochlear Ribbon Synapses. J Neurosci.

[36] Liu HS, Jan MS, Chou CK, Chen PH, Ke NJ (1999). Is green fluorescent protein toxic to the living cells?. Biochem Biophys Res Commun.

[37] Taghizadeh RR, Sherley JL (2008). CFP and YFP, but not GFP, provide stable fluorescent marking of rat hepatic adult stem cells. J Biomed Biotechnol.

[38] Huang WY, Aramburu J, Douglas PS, Izumo S (2000). Transgenic expression of green fluorescence protein can cause dilated cardiomyopathy. Nat Med.

[39] Chen H (2018). AAV-mediated NT-3 overexpression protects cochleae against noise-induced synaptopathy. Gene Ther.

[40] Royo NC (2008). Specific AAV serotypes stably transduce primary hippocampal and cortical cultures with high efficiency and low toxicity. Brain Res.

[41] Hirsch ML (2011). Viral single-strand DNA induces p53-dependent apoptosis in human embryonic stem cells. PLoS One.

[42] Ulusoy A, Sahin G, Bjorklund T, Aebischer P, Kirik D (2009). Dose optimization for long-term rAAV-mediated RNA interference in the nigrostriatal projection neurons. Mol Ther.

